# The association between shift work, shift work sleep disorders and premature ejaculation in male workers

**DOI:** 10.1186/s12889-024-19141-1

**Published:** 2024-07-03

**Authors:** Zhenming Zheng, Jiashan Pan, Zhimin Chen, Pan Gao, Jingjing Gao, Hui Jiang, Xiansheng Zhang

**Affiliations:** 1 Department of Urology, the First Affiliated Hospital of Anhui Medical University, No. 218 Jixi Road, Shushan District, Hefei, Anhui Province 230022 China; 2 Institute of Urology, Anhui Medical University, Hefei, Anhui China; 3 Anhui Province Key Laboratory of Urological and Andrological Diseases Research and Medical Transformation, Hefei, Anhui China; 4 Andrology Center, Peking University First Hospital, No. 8 Xishiku Street, Xicheng District, Beijing, 100034 China

**Keywords:** Shift work, Shift work sleep disorder, Premature ejaculation, Risk factors, Predictive model

## Abstract

**Objective:**

Shift work and Shift Work Sleep Disorder (SWSD) are known to affect the secretion of several neurotransmitters and hormones associated with premature ejaculation (PE). However, their specific influence on the regulation of male ejaculation remains unclear. This study explores the relationship between shift work, SWSD, and PE.

**Methods:**

From April to October 2023, a cross-sectional survey was conducted across five regions of China to explore the work schedules, sleep quality, and sexual function of male workers. Participants' sleep quality was evaluated using a validated SWSD questionnaire, and their erectile function and ejaculatory control were assessed with the International Inventory of Erectile Function (IIEF-5) scores and Premature Ejaculation Diagnostic Tool (PEDT) scores, respectively. Univariate and multivariate linear regression analyses were employed to identify risk factors associated with PE. Confounders were controlled using multiple regression models, and clinical prediction models were developed to predict PE onset and assess the contribution of risk factors.

**Results:**

The study included 1239 eligible participants, comprising 840 non-shift workers and 399 shift workers (148 with SWSD and 251 without SWSD). Compared to non-shift working males, those involved in shift work (β 1.58, 95% CI 0.75 – 2.42, *p* < 0.001) and those suffering from SWSD (β 2.86, 95% CI 1.86 – 3.85, *p* < 0.001) they had significantly higher PEDT scores. Additionally, we identified daily sleep of less than six hours, depression, anxiety, diabetes, hyperlipidemia, frequent alcohol consumption (more than twice a week), and erectile dysfunction as risk factors for PE. The predictive model for PE demonstrated commendable efficacy.

**Conclusion:**

Both shift work and SWSD significantly increase the risk of premature ejaculation, with the risk magnifying in tandem with the duration of shift work. This study reveals the potential impact of shift work and SWSD on PE and provides new theoretical foundations for the risk assessment and prevention of this condition.

**Supplementary Information:**

The online version contains supplementary material available at 10.1186/s12889-024-19141-1.

## Introduction

With the development of industrialization, an increasing number of factories have adopted a 24-h shift work system to enhance productivity. Additionally, certain professions such as nursing and policing necessitate shift schedules to ensure 24-h staffing. Nearly one-fifth of employees worldwide are engaged in some form of shift work [1, 2]. Non-standard shift work is defined as starting before 07:00 or after 14:00, rotated, or regularly including hours outside the standard 07:00 to 18:00 workday [3, 4]. Frequent transitions between night and day shifts can disrupt circadian rhythms, a considerable proportion of shift workers suffer from shift work sleep disorder (SWSD), characterized by excessive sleepiness, insomnia, or a combination of both [5]. A large cross-sectional survey from China found that 19.9% of shift workers experienced SWSD [6]. With the increasing prevalence of shift work, the substantial population with SWSD has become a significant public health concern [7].

Shift work and the SWSD have multifaceted negative effects on physical and psychological health, exacerbating risks of anxiety, depression, cardiovascular diseases, stroke, endocrine dysfunctions, and even elevating the likelihood of maladies such as breast cancer [7–9]. In males, the repercussions of shift work and sleep disturbances are widespread, encompassing an increased risk of hypogonadism and diminished testosterone levels [10], with potential detriments to erectile function [11] and reproductive capacity [12]. It is worth noting that these diseases and health conditions, which are strongly associated with shift work and sleep disorders, are also strongly associated with premature ejaculation [13–16]. However, there is a dearth of reports on the impact of shift work and sleep disturbances on male ejaculatory regulation and premature ejaculation. Previous studies indicate that the onset of premature ejaculation is influenced by a variety of neurotransmitters (such as serotonin, dopamine, γ-aminobutyric acid) and hormones (such as testosterone) [17–20], which are, in turn, regulated by sleep rhythms and duration [21, 22]. Therefore, we postulate that shift work and sleep disturbances could potentially indirectly influence the occurrence of premature ejaculation by affecting the secretion of central neurotransmitters and increasing the risk of other associated diseases.

To explore the association between shift work, SWSD, and premature ejaculation, this study conducted a representative sampling survey across the South, North, East, West, and Central China regions—the research aimed to investigate the impact of shift work and SWSD on premature ejaculation. Additionally, based on various risk factors a clinical prediction model for premature ejaculation was constructed, providing important references for the clinical identification of risk factors associated with premature ejaculation and guidance on its prevention and treatment.

## Methods

This study was conducted in accordance with the World Medical Association’s Declaration of Helsinki: Ethical Principles for Medical Research Involving Human Subjects [23]. The participants provided their written informed consent to participate in this study. The Ethics Committee of the First Affiliated Hospital of Anhui Medical University reviewed and approved the study (No. Pj2024-03–38).

### Study design and participants

Initiated by the China Sexology Association, a nationally representative sampling survey was conducted across two cities in each of the five geographical regions of China: East (Shanghai, Hefei), West (Chengdu, Chongqing), South (Guangzhou, Dongguan), North (Beijing, Shijiazhuang), and Central (Wuhan, Changsha). The investigation focused on the work schedules, sleep quality, and sexual function of male workers. Data collection was carried out in community streets by urologists, graduate students in urology, and community doctors from April to October 2023. A secure and reliable electronic questionnaire collection platform (Wenjuan Star, Changsha Ranxing Information Technology, https://www.wjx.cn) was used to facilitate anonymous online survey participation. Participants filled out the questionnaire by scanning its QR code via WeChat and received professional assistance from urologists, graduate students in urology, and community doctors to ensure accurate comprehension of the questions. A pilot study involving 10% of the total sample was conducted beforehand to ensure the questionnaire’s practicability.

### Data collection procedure and data quality control

The questionnaire compiled data on age, height, weight, education, marital status, hypertension, diabetes, hyperlipidemia, smoking, alcohol consumption, work shifts, sleep quality, and sexual function. According to previous studies, a non-standard shift is defined as one that starts before 07:00 or after 14:00, involves rotation, or is frequently worked outside the standard shift period (07:00–18:00 h). Work within the standard shift period, but frequently requiring overtime of several hours, is not recognized as a non-standard shift [3, 4]. The diagnostic algorithm and questionnaire content for SWSD is based on the International Classification of Sleep Disorders-3 (ICSD-3) diagnostic criteria and questionnaires developed by Barger LK et al. [3, 24]. Erectile function and ejaculation timing were evaluated using the International Inventory of Erectile Dysfunction (IIEF-5) scores and Premature Ejaculation Diagnostic Tool (PEDT) scores, respectively, classifying an IIEF-5 score ≤ 21 as erectile dysfunction and a PEDT score ≥ 11 as indicative of premature ejaculation. The General Anxiety Disorder-7 (GAD-7) and Patient Health Questionnaire-9 (PHQ-9) scored for anxiety and depression with scores ≥ 5 suggesting the presence of anxiety or depression, respectively. Detailed variable names and the questionnaire are provided in Supplementary Material 1.

After collecting the data, we performed quality control on the questionnaire data based on the following inclusion and exclusion criteria to eliminate surveys that did not meet the standards. Inclusion criteria were: (1) males aged 18 to 60; (2) individuals with a stable spouse or girlfriend engaging in regular sexual activity; (3) no severe underlying medical conditions, such as serious spinal cord injury, paraplegia, or advanced malignant tumors. Exclusion criteria included: (1) persons with psychiatric anomalies; (2) respondents whose survey answers were contradictory or who provided incomplete data; (3) completion of surveys in under three minutes, which were considered to reflect insincerity in response.

### Statistical analysis

Data were processed and analyzed using R version 4.3.0 and SPSS version 25.0 for Windows (IBM Corporation, Armonk, New York, USA). Continuous data conforming to a normal distribution were presented as mean ± standard deviation (SD) and analyzed using Student’s t-test or Mann–Whitney test for inter-group differences. Categorical variables were described using frequency counts and percentages and analyzed using Pearson’s chi-square test or Fisher’s exact test. Univariate and multivariate logistic regression analysis was used to identify risk factors associated with premature ejaculation, and multiple regression models after controlling for confounders were used to further assess the association between shift work and SWSD and premature ejaculation. *P* < 0.05 was considered statistically significant.

Furthermore, a nomogram was developed to predict the risk of premature ejaculation among men of different work shifts. The model was constructed by randomly dividing all participants into a training set and a validation set in a ratio of 6:4. The nomogram’s specificity and sensitivity were assessed by plotting the ROC curve and calculating the Area Under the Curve (AUC). Calibration plots and Decision Curve Analysis (DCA) were drawn to validate the model’s reliability.

## Results

### Characteristics of the study population

**Fig. 1:**
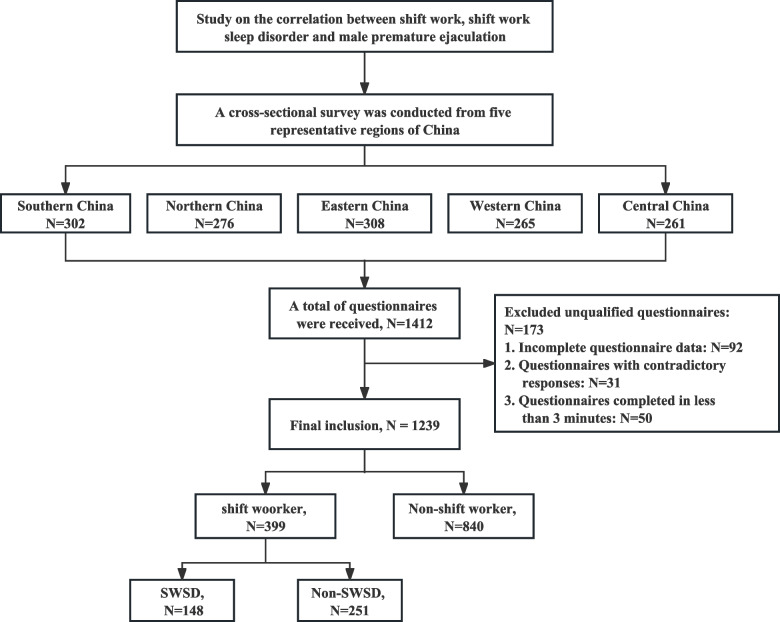
Flow chart of enrollment for this cross-sectional study

A total of 1412 questionnaire responses were obtained. After excluding ineligible data, data from 1239 male participants were included in the final analysis. Of these, 840 were non-shift workers, and 399 were shift workers—among which 148 suffered from SWSD, while 251 did not have SWSD. The detailed inclusion process is depicted in Fig. 1. We compared the clinical characteristics, sleep quality, and sexual function between shift workers and non-shift workers, as well as between males with SWSD and those without SWSD. The results revealed that, compared to non-shift working males, shift working males had a higher proportion of young individuals under the age of 45, higher Body Mass Index (BMI), and higher prevalence of hypertension, diabetes, hyperlipidemia, anxiety, and depression. They also had higher Premature Ejaculation Diagnostic Tool (PEDT) scores, lower International Index of Erectile Function-5 (IIEF-5) scores, and a greater percentage reporting insufficient sleep and working times exceeding 8 h (Table 1). These conditions were found to be more severe among shift working males with SWSD (Table 2).

**Table 1:** Demographic and clinical characteristics of shift and non-shift male workers

Variable	Total (*n* = 1239)	Non-shift workers (*n* = 840)	Shift workers (*n* = 399)	*p*-Value
**Age (yr.)**				0.046
18–44	1074 (86.68)	717 (85.36)	357 (89.47)	
45–60	165 (13.32)	123 (14.64)	42 (10.53)	
**BMI (kg/m**^**2**^**)**				0.012
< 24	576 (46.49)	411 (48.93)	165 (41.35)	
≥ 24	663 (53.51)	429 (51.07)	234 (58.65)	
**Education**				0.236
Primary school	36 (2.91)	21 (2.50)	15 (3.76)	
Secondary school	393 (31.72)	255 (30.36)	138 (34.59)	
Undergraduate in Regular HEIs	681 (54.96)	474 (56.43)	207 (51.88)	
Postgraduates	129 (10.41)	90 (10.71)	39 (9.77)	
**Marital Status**				0.467
Married	1134 (91.53)	768 (91.43)	366 (91.73)	
Divorced	57 (4.6)	42 (5.00)	15 (3.76)	
Unmarried	48 (3.87)	30 (3.57)	18 (4.51)	
**Hypertension**				0.007
No	1080 (87.17)	747 (88.93)	333 (83.46)	
Yes	159 (12.83)	93 (11.07)	66 (16.54)	
**Diabetes**				< .001
No	1140 (92.01)	801 (95.36)	339 (84.96)	
Yes	99 (7.99)	39 (4.64)	60 (15.04)	
**Hyperlipidemia**				0.004
No	1131 (91.28)	780 (92.86)	351 (87.97)	
Yes	108 (8.72)	60 (7.14)	48 (12.03)	
**Smoking**				0.028
Non-smoker	567 (45.76)	405 (48.21)	162 (40.60)	
Current	507 (40.92)	333 (39.64)	174 (43.61)	
Past	165 (13.32)	102 (12.14)	63 (15.79)	
**Drinking**				0.361
Never	306 (24.7)	213 (25.36)	93 (23.31)	
Frequently	147 (11.86)	105 (12.50)	42 (10.53)	
Seldom	786 (63.44)	522 (62.14)	264 (66.17)	
**Sleep hours/day**				< .001
6–8 h	990 (79.9)	717 (85.36)	273 (68.42)	
< 6 h	186 (15.01)	78 (9.29)	108 (27.07)	
> 8 h	63 (5.08)	45 (5.36)	18 (4.51)	
**Work hours/day**				< .001
≤ 8h	651 (52.54)	522 (62.14)	129 (32.33)	
> 8 h	588 (47.46)	318 (37.86)	270 (67.67)	
**Midday nap**				< .001
Yes	741 (59.81)	552 (65.71)	189 (47.37)	
No	498 (40.19)	288 (34.29)	210 (52.63)	
**Depression**				< .001
No	912 (73.61)	729 (86.79)	183 (45.86)	
Yes	327 (26.39)	111 (13.21)	216 (54.14)	
**Anxiety**				< .001
No	930 (75.06)	723 (86.07)	207 (51.88)	
Yes	309 (24.94)	117 (13.93)	192 (48.12)	
**PEDT score**	9.00 (5.50—13.00)	7.00 (3.50—11.00)	12.00 (10.00—14.00)	< .001
**IIEF-5 score**	20.00 (13.00—22.00)	22.00 (16.00—23.00)	18.00 (14.00—20.00)	< .001

Education categories include: Primary school, includes primary school or not completed primary school; Secondary school, inclusive of junior, senior, and vocational school; Undergraduate in Regular HEIs, includes normal courses and short-cycle courses; Postgraduates, includes doctor´s degree and master´s degree. Statistical descriptions of non-normally distributed continuous count data were conducted using median (Q1, Q3), while categorical variables were presented as counts (%). A significance level of *P* < 0.05 was considered statistically significant

*Abbreviations*: *IIEF-5* International Index of Erectile Function-5, *PEDT* premature ejaculation diagnostic tool, *BMI* Body Mass Index

**Table 2:** Demographic and clinical characteristics of SWSD and non-SWSD shift workers

Variable	Total (*n* = 399)	Non-SWSD (*n* = 251)	SWSD (*n* = 148)	*p*-Value
**Age (yr.)**				0.026
18–44	357 (89.47)	218 (86.85)	139 (93.92)	
45–60	42 (10.53)	33 (13.15)	9 (6.08)	
**BMI (kg/m**^**2**^**)**				0.018
< 24	165 (41.35)	115 (45.82)	50 (33.78)	
≥ 24	234 (58.65)	136 (54.18)	98 (66.22)	
**Education**				0.005
Primary school	15 (3.76)	15 (5.98)	0 (0.00)	
Secondary school	138 (34.59)	92 (36.65)	46 (31.08)	
Undergraduate in Regular HEIs	207 (51.88)	124 (49.40)	83 (56.08)	
Postgraduates	39 (9.77)	20 (7.97)	19 (12.84)	
**Marital Status**				0.009
Married	366 (91.73)	224 (89.24)	142 (95.95)	
Divorced	15 (3.76)	15 (5.98)	0 (0.00)	
Unmarried	18 (4.51)	12 (4.78)	6 (4.05)	
**Hypertension**				< .001
No	333 (83.46)	228 (90.84)	105 (70.95)	
Yes	66 (16.54)	23 (9.16)	43 (29.05)	
**Diabetes**				< .001
No	339 (84.96)	230 (91.63)	109 (73.65)	
Yes	60 (15.04)	21 (8.37)	39 (26.35)	
**Hyperlipidemia**				< .001
No	351 (87.97)	240 (95.62)	111 (75.00)	
Yes	48 (12.03)	11 (4.38)	37 (25.00)	
**Smoking**				< .001
Non-smoker	162 (40.6)	95 (37.85)	67 (45.27)	
Current	174 (43.61)	130 (51.79)	44 (29.73)	
Past	63 (15.79)	26 (10.36)	37 (25.00)	
**Drinking**				0.035
Never	93 (23.31)	48 (19.12)	45 (30.41)	
Frequently	42 (10.53)	27 (10.76)	15 (10.14)	
Seldom	264 (66.17)	176 (70.12)	88 (59.46)	
**Sleep hours/day**				< .001
6–8 h	273 (68.42)	183 (72.91)	90 (60.81)	
< 6 h	108 (27.07)	53 (21.12)	55 (37.16)	
> 8 h	18 (4.51)	15 (5.98)	3 (2.03)	
**Work hours/day**				0.009
≤ 8h	129 (32.33)	93 (37.05)	36 (24.32)	
> 8 h	270 (67.67)	158 (62.95)	112 (75.68)	
**Midday nap**				0.101
Yes	189 (47.37)	111 (44.22)	78 (52.70)	
No	210 (52.63)	140 (55.78)	70 (47.30)	
**Shift Work Years**				0.006
< 1 years	135 (33.83)	99 (39.44)	36 (24.32)	
1–3 years	136 (34.09)	82 (32.67)	54 (36.49)	
> 3 years	128 (32.08)	70 (27.89)	58 (39.19)	
**Depression**				< .001
No	183 (45.86)	163 (64.94)	20 (13.51)	
Yes	216 (54.14)	88 (35.06)	128 (86.49)	
**Anxiety**				< .001
No	207 (51.88)	158 (62.95)	49 (33.11)	
Yes	192 (48.12)	93 (37.05)	99 (66.89)	
**PEDT score**	9.00 (5.50—13.00)	7.00 (3.50—11.00)	12.00 (10.00—14.00)	< .001
**IIEF-5 score**	20.00 (13.00—22.00)	22.00 (16.00—23.00)	18.00 (14.00—20.00)	< .001

Statistical descriptions of non-normally distributed continuous count data were conducted using median (Q1, Q3), while categorical variables were presented as counts (%). A significance level of *P* < 0.05 was considered statistically significant

*Abbreviations*: *SWSD* Shift work sleep disorder, *BMI* Body Mass Index, *IIEF-5* International Index of Erectile Function-5, *PEDT* premature ejaculation diagnostic tool

### The association between shift work, SWSD and premature ejaculation

The results of univariate and multivariate linear regression analyses on premature ejaculation indicate (as shown in Table 3) that compared to non-shift-working males, men engaged in shift work are more predisposed to premature ejaculation, with a significant increase in PEDT score (β 1.58, 95% CI 0.75—2.42, *p* < 0.001). For males with Shift Work Sleep Disorder (SWSD), the risk of premature ejaculation is further elevated, as reflected by an increased PEDT score (β 2.86, 95% CI 1.86—3.85, *p* < 0.001). Subgroup analysis based on the duration of shift work reveals that less than one year of shift work does not affect the occurrence of premature ejaculation, whereas the prevalence significantly rises with more than one year of shift work, and even more so after three years. Moreover, our findings suggest that less than six hours of sleep per day, depression, anxiety, diabetes, hyperlipidemia, frequent alcohol consumption (more than twice per week), and erectile dysfunction are risk factors for premature ejaculation. In addition, a higher percentage of premature ejaculation occurs in men older than 45 and overweight (Table 4).

**Table 3:** Univariate and multivariate linear regression analyses of PEDT scores in shift work and SWSD male workers

Variables	Univariate		Multivariate	
	**β (95%CI)**	***p*-value**	**β (95%CI)**	***p*-Value**
**Shift work**				
No	0.00 (Reference)		0.00 (Reference)	
Yes	3.89 (3.31 ~ 4.46)	< .001	1.58 (0.75 ~ 2.42)	**< .001**
**SWSD**				
No	0.00 (Reference)		0.00 (Reference)	
Yes	5.08 (4.20 ~ 5.96)	< .001	2.86 (1.86 ~ 3.85)	**< .001**
**Shift work years**				
0	0.00 (Reference)		0.00 (Reference)	
< 1 years	2.27 (1.40 ~ 3.14)	< .001	-0.62 (-1.62 ~ 0.38)	0.226
1–3 years	5.14 (4.28 ~ 6.01)	< .001	1.45 (0.77 ~ 2.14)	**< .001**
> 3 years	4.26 (3.37 ~ 5.15)	< .001	1.93 (1.03 ~ 3.45)	**< .001**
**Sleep hours/day**				
6–8 h	0.00 (Reference)		0.00 (Reference)	
< 6 h	3.86 (3.10 ~ 4.63)	< .001	1.66 (0.95 ~ 2.36)	**< .001**
> 8 h	-2.92 (-4.17 ~ -1.67)	< .001	-0.27 (-1.39 ~ 0.16)	0.321
**Work hours/day**				
≤ 8h	0.00 (Reference)		0.00 (Reference)	
> 8 h	2.88 (2.33 ~ 3.43)	< .001	1.06 (0.55 ~ 1.57)	**< .001**
**Midday nap**				
Yes	0.00 (Reference)		0.00 (Reference)	
No	0.88 (0.30 ~ 1.46)	0.003	0.56 (0.05 ~ 1.07)	**0.030**
**Depression**				
No	0.00 (Reference)		0.00 (Reference)	
Yes	5.22 (4.64 ~ 5.81)	< .001	1.32 (0.71 ~ 1.93)	**< .001**
**Anxiety**				
No	0.00 (Reference)		0.00 (Reference)	
Yes	3.88 (3.25 ~ 4.50)	< .001	1.29 (0.68 ~ 1.91)	**< .001**
**Erectile dysfunction**				
No	0.00 (Reference)		0.00 (Reference)	
Yes	3.16 (2.53 ~ 3.79)	< .001	3.06 (1.43 ~ 4.69)	**< .001**
**Age (yr.)**				
18–30	0.00 (Reference)		0.00 (Reference)	
31–45	-0.87 (-1.49 ~ -0.25)	0.006	0.14 (-0.43 ~ 0.71)	0.640
46–60	-3.12 (-4.07 ~ -2.17)	< .001	0.45 (-0.34 ~ 1.23)	0.262
**BMI (kg/m**^**2**^**)**				
< 18.5	0.00 (Reference)		0.00 (Reference)	
18.5–24	-1.85 (-2.71 ~ -0.99)	< .001	-0.48 (-1.06 ~ 0.09)	0.101
> 24	-1.67 (-2.32 ~ -1.02)	< .001	-0.83 (-2.02 ~ 0.37)	0.175
**Education**				
Primary school	0.00 (Reference)			
Secondary school	1.27 (-0.47 ~ 3.01)	0.153		
Undergraduate in Regular HEIs	-0.25 (-1.96 ~ 1.46)	0.775		
Postgraduates	-0.90 (-2.78 ~ 0.99)	0.351		
**Marital Status**				
Married	0.00 (Reference)		0.00 (Reference)	
Divorced	-1.44 (-2.81 ~ -0.07)	0.040	0.79 (-0.44 ~ 2.02)	0.208
Unmarried	1.10 (-0.39 ~ 2.58)	0.149	-0.34 (-1.13 ~ 0.46)	0.411
**Hypertension**				
No	0.00 (Reference)		0.00 (Reference)	
Yes	1.04 (0.19 ~ 1.90)	0.017	0.90 (-0.08 ~ 1.88)	0.073
**Diabetes**				
No	0.00 (Reference)		0.00 (Reference)	
Yes	3.20 (2.16 ~ 4.25)	< .001	1.10 (0.17 ~ 2.03)	**0.020**
**Hyperlipidemia**				
No	0.00 (Reference)		0.00 (Reference)	
Yes	1.97 (0.96 ~ 2.98)	< .001	1.15 (0.22 ~ 2.09)	**0.015**
**Smoking**				
Non-smoker	0.00 (Reference)		0.00 (Reference)	
Current	1.37 (0.48 ~ 2.26)	0.003	0.94 (0.17 ~ 1.71)	**0.017**
Past	0.09 (-0.52 ~ 0.71)	0.767	0.28 (-0.26 ~ 0.82)	0.314
**Drinking**				
Never	0.00 (Reference)		0.00 (Reference)	
Frequently	0.84 (0.16 ~ 1.52)	0.016	0.87 (0.28 ~ 1.46)	**0.004**
Seldom	0.15 (-0.86 ~ 1.16)	0.769	0.65 (-0.24 ~ 1.55)	0.154

GAD-7 scores ≥ 5 were considered to be anxiety; PHQ-9 scores ≥ 5 were considered to be depression. IIEF-5 scores < 22 were considered to have erectile dysfunction. *P* < 0.05 was considered to be a statistically significant difference

*Abbreviations*: *β* Beta value, *CI* Confidence Interval, *SWSD* Shift work sleep disorder, *BMI* Body Mass Index, *IIEF-5* International Index of Erectile Function-5, *PEDT* premature ejaculation diagnostic tool

**Table 4:** Comparison of clinical characteristics between participants with and without premature ejaculation

Variable	Total	Non-PE	PE	*p*-Value
	**(*n* = 1239)**	**(*n* = 950)**	**(*n* = 289)**	
**Age (yr.)**				< .001
18–44	1074 (86.68)	845 (88.95)	229 (79.24)	
45–60	165 (13.32)	105 (11.05)	60 (20.76)	
**BMI (kg/m**^**2**^**)**				0.003
< 24	576 (46.49)	464 (48.84)	112 (38.75)	
≥ 24	663 (53.51)	486 (51.16)	177 (61.25)	
**Sleep hours/day**				< .001
6–8 h	990 (79.90)	800 (84.21)	190 (65.74)	
< 6 h	186 (15.01)	87 (9.16)	99 (34.26)	
> 8 h	63 (5.08)	63 (6.63)	0 (0.00)	
**Work hours/day**				< .001
≤ 8h	651 (52.54)	553 (58.21)	98 (33.91)	
> 8 h	588 (47.46)	397 (41.79)	191 (66.09)	
**SWSD**				< .001
Non-shift work	840 (67.80)	686 (72.21)	154 (53.29)	
No	148 (11.95)	80 (8.42)	68 (23.53)	
Yes	251 (20.26)	184 (19.37)	67 (23.18)	
**Depression**				< .001
No	912 (73.61)	800 (84.21)	112 (38.75)	
Yes	327 (26.39)	150 (15.79)	177 (61.25)	
**Anxiety**				< .001
No	930 (75.06)	783 (82.42)	147 (50.87)	
Yes	309 (24.94)	167 (17.58)	142 (49.13)	
**Erectile dysfunction**				< .001
No	918 (74.09)	770 (81.05)	148 (51.21)	
Yes	321 (25.91)	180 (18.95)	141 (48.79)	

Premature Ejaculation (PE) is defined based on the Premature Ejaculation Diagnostic Tool (PEDT) score, with ≥ 11 being considered as PE and < 11 as non-PE. Erectile Dysfunction (ED) is defined based on the International Index of Erectile Function-5 (IIEF-5) score, with < 22 indicating the presence of erectile dysfunction and ≥ 22 signifying normal erectile function. Categorical variables were presented as counts (%)

*Abbreviations*: *SWSD* Shift Work Sleep Disorder, *BMI* Body Mass Index

To control for the influence of confounding factors and further evaluate the association between shift work, SWSD, and premature ejaculation, multiple regression models adjusting for various variables were employed. The first model did not adjust for any variables, the second model adjusted for clinical baseline characteristics excluding sleep and shift work-related variables, and the third model included adjustments for all variables. The results from all three models consistently showed that, compared to men who do not work shifts, men who do engage in shift work had higher premature ejaculation diagnostic scores (Model 1, β 3.89, 95% CI 3.31—4.46, *p* < 0.001; Model 2, β 1.22, 95% CI 0.62—1.83, *p* < 0.001; Model 3, β 1.56, 95% CI 0.74—2.39, *p* < 0.001). Similarly, men with SWSD had higher premature ejaculation diagnostic scores than those without SWSD (Model 1, β 5.08, 95% CI 4.20—5.96, *p* < 0.001; Model 2, β 4.15, 95% CI 3.19—5.12, *p* < 0.001; Model 3, β 4.03, 95% CI 3.09—4.98, *p* < 0.001) (as depicted in Table 5).

**Table 5:** Assessment of the association between shift work, SWSD, and PEDT scores using multiple regression models controlling for confounding factors

Variables	Model 1		Mode 2		Model 3	
	**β (95%CI)**	***P***	**β (95%CI)**	***P***	**β (95%CI)**	***P***
**Shift work**						
No	0.00 (Reference)		0.00 (Reference)		0.00 (Reference)	
Yes	3.89 (3.31 ~ 4.46)	**< .001**	1.22 (0.62 ~ 1.83)	**< .001**	1.56 (0.74 ~ 2.39)	**< .001**
**SWSD**						
No	0.00 (Reference)		0.00 (Reference)		0.00 (Reference)	
Yes	5.08 (4.20 ~ 5.96)	**< .001**	4.15 (3.19 ~ 5.12)	**< .001**	4.03 (3.09 ~ 4.98)	**< .001**

Model 1: crude

Model 2: adjusted for Age, BMI, Education, Marital Status, Hypertension, Diabetes, Hyperlipidemia, Smoking, Drinking, PHQ-9, GAD-7, IIEF-5

Model 3: adjusted for adjusted for Age, BMI, Education, Marital Status, Hypertension, Diabetes, Hyperlipidemia, Smoking, Drinking, PHQ-9, GAD-7, IIEF-5. Sleep hours/day, Work hours/day, Midday Nap, Shift work years

*Abbreviations*: *SWSD* Shift work sleep disorder, *β* Beta value, *CI* Confidence Interval, *PEDT* Premature Ejaculation Diagnostic Tool

### Performance of prediction model

**Fig. 2:**
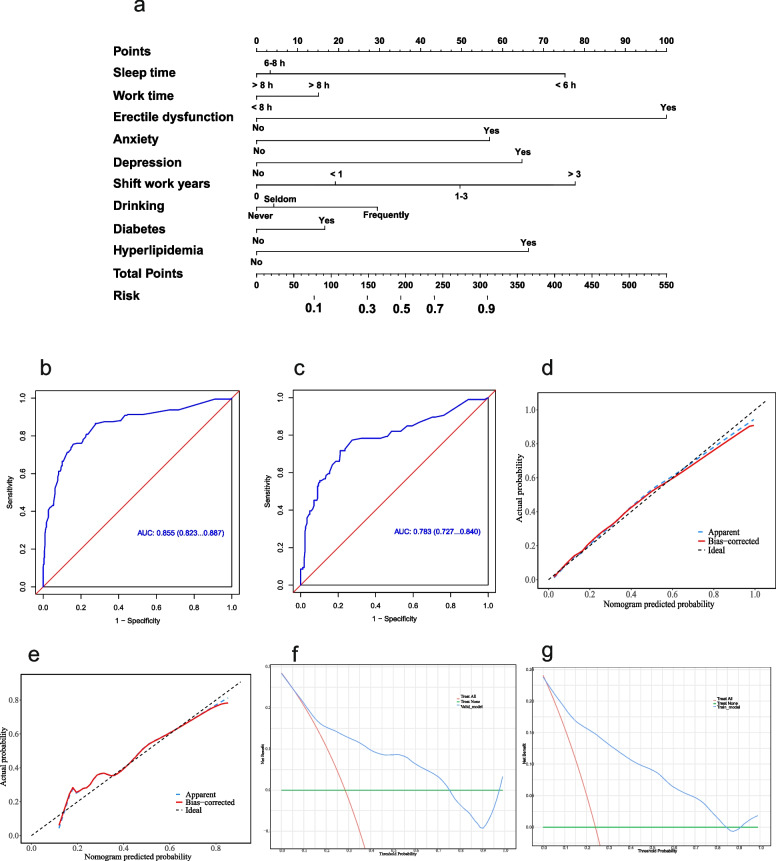
Nomogram construction and validation for predicting the risk of premature ejaculation. **a** A nomogram was developed to predict the risk of premature ejaculation onset. **b**, **c** Receiver Operating Characteristic (ROC) curves illustrate good sensitivity and specificity of the predictive model with an area under the curve (AUC) of 0.855 for the training set and 0.783 for the validation set. **d**, **e** Calibration plots demonstrate high predictive accuracy, with close alignment between the actual and predicted probabilities of premature ejaculation. **f**, **g** Decision curve analysis indicates the substantial clinical utility of the nomogram by evaluating the net benefits at different threshold probabilities

To more precisely quantify the contributory impact of each risk factor on the onset of premature ejaculation, we integrated risk factors identified through multifactorial regression analysis with clinical realities and constructed a nomogram including variables such as daily sleep hours, daily work hours, erectile dysfunction, anxiety, depression, years of shift work, alcohol consumption, diabetes, and hyperlipidemia, for the prospective forecasting of premature ejaculation incidents. Clinicians may utilize this nomogram to assign risk scores to patients, thereby assessing their risk of developing premature ejaculation. The model has been validated and demonstrated commendable sensitivity and specificity (with an Area Under the Curve, AUC, of 0.855 in the training set and 0.727 in the validation set). Calibration and decision curves further substantiate the model’s reliability (Fig. 2).

## Discussion

This large-scale survey explored the association between shift work, SWSD, and the risk of premature ejaculation (PE) among Chinese male shift workers. The results suggest that men engaged in shift work, particularly those suffering from SWSD, are more likely to report issues of PE. Combining the results of this study with findings from previous studies, we hypothesize that metabolic alterations, causing mental and psychological disorders, and affecting central neurotransmitter secretion may be potential pathways through which shift work affects premature ejaculation.

Shift work-related metabolic alterations, this study found that men who worked shift work and suffered from SWSD were more likely to have diabetes, hyperlipidemia, and be overweight compared to non-shift working men and that these problems were more severe in shift men with comorbid premature ejaculation. This may be related to shift work workers' irregular diet and frequent late-night snacking. In addition, the altered circadian rhythms and irregular diets associated with shift work and SWSD affect insulin and glucagon secretion, disturbing metabolic homeostasis and increasing the risk of cardiovascular disease and metabolic disorders [16, 25, 26], which are risk factors for premature ejaculation [18]. Our survey indicates that there are fewer middle-aged and older men in shift work and suffering from SWSD, suggesting that younger individuals are the main workforce engaged in shift work; however, the health of these younger workers should also be a matter of concern.

Shift work-related psychiatric and psychological disorders, our findings suggest that men who work shifts and suffer from SWSD are more likely to experience depression and anxiety. Previous studies have also found that shift work and SWSD-induced sleep problems can increase the risk of anxiety and depression [27]. Anxiety and depression are risk factors for premature ejaculation [15]. In addition, lifestyle changes due to shift work may also lead to sleep desynchronization and sexual irregularities between partners, further increasing the risk of premature ejaculation.

Shift work-associated central neurotransmitter alterations, circadian rhythm changes, and sleep disturbances caused by shift work and SWSD may also interfere with the secretion of a variety of neurotransmitters closely associated with premature ejaculation (including 5-HT, dopamine, GABA, and glutamate) [28, 29]. Moreover, these neurotransmitters also play an important role in sleep regulation, and thus, there may be a mutual regulatory relationship between them [21, 30]. In addition, shift work and SWSD may also affect steroid hormone changes in the body [31], increase the risk of hypogonadism [10], and even impair erectile function in men [11]. These biological changes may directly or indirectly affect the development of premature ejaculation.

Ssleep duration and the duration of engaging in shift work are also significant factors affecting PE. Our study also found that compared with non-shift men, shift and SWSD men had shorter average daily sleep duration, and men who slept less than six hours per day had a significantly increased risk of PE. A representative national survey from the United States found that nocturnal workers had shorter sleep durations compared to daytime workers and were more likely to experience sleep disorders [32]. Additionally, our research found that the risk of PE increases correspondingly with the duration of shift work engagement, with the differences manifesting after one year of shift work. This may imply an inability of males to adapt to the circadian changes imposed by shift work, as the risk for various factors associated with PE, such as hypertension, diabetes, dyslipidemia, endocrine and metabolic disorders, and psychological disturbances, also seems to increase with the duration of shift work [7]. Therefore, we recommend that shift workers ensure adequate sleep, maintain regular eating habits, and foster a positive mental outlook to mitigate the occurrence of SWSD. Should symptoms of SWSD arise, they should promptly adjust their sleeping and working schedules, seeking medical assistance when necessary, to reduce the overall physical and psychological damage induced by shift work. Overall, shift work and SWSD may influence the occurrence of premature ejaculation through direct or indirect pathways.

Admittedly, there are some limitations to this study; firstly, we used the SWSD questionnaire to assess participants’ sleep quality, and although this questionnaire has been validated in several studies [1, 3, 32], it may still lack accuracy compared to objective polysomnographic monitoring results. Second, our study did not take into account the potential impact of chemical and physical factors to which we are exposed during work and differences in shift work periods on premature ejaculation. Finally, this was a large-scale cross-sectional survey study, and more well-designed prospective studies are needed in the future to further validate our findings.

## Conclusion

This study has unveiled the adverse impact of shift work and SWSD on PE, with risks that escalate as the duration of shift work increases. These findings afford a new perspective for further understanding the etiology of PE and for guiding future research directions. Nevertheless, the exact mechanisms by which shift work and sleep disorders affect PE require additional exploration.

### Supplementary Information

Supplementary Material 1.

## Data Availability

No datasets were generated or analysed during the current study.
