# Supplementary material for: The association between shift work, shift work sleep disorders and premature ejaculation in male workers
Source: BMC Public Health. 2024 Jul 3;24:1772. doi: 10.1186/s12889-024-19141-1 (PMC11223354; doi:10.1186/s12889-024-19141-1)
Supplement: Supplementary file 1 — Supplementary Material 1. [file 12889_2024_19141_MOESM1_ESM.docx]

**Questionnaire on Sleep and Sexual Function in Day and Shift Work Men**

Dear Male Friends,

      With the progress of industrialization, many factories have adopted a 24-hour rotating shift system. In addition, there are some special positions that require a 24-hour rotating shift due to the nature of the work. The population working night shifts or rotating shifts is becoming increasingly large. As an andrologists, during our long-term outpatient work, we have found that changes in male sexual function may be related to the timing of day shifts, night shifts, or rotating shifts. To care for men's health and further explore the relationship between male sexual function and sleep, the Chinese Society of Sexology is organizing a large-scale online survey titled "Sleep and Sexual Function in Male Workers on Day Shifts or Rotating Shifts". This questionnaire survey will be conducted anonymously, and we will strictly protect the privacy of each participant. Thank you for your participation!

**Part I. Basic characteristics**

1. Your age is ( ) years

2 Your height is ( ) cm

Your weight is ( ) kg

3. Your level of education is ( ).

| A. Elementary school or below |
| --- |
| B. Secondary school (including middle school, high school and junior college) |
| C. Universities (including junior colleges and undergraduate programs) |
| D. Postgraduate students (including master's degree and doctoral degree) |

4. Your city ( )

5. Your marital status is ( ) [Single choice]

| A. Unmarried |
| --- |
| B. Married, currently in a marriage |
| D. Divorced |

6. Do you suffer from hypertension ? ( )

| A. is |
| --- |
| B. No |

How many years have you suffered from hypertension?( )

Dependent on the first option of question 6.

7. Do you suffer from diabetes? ( )

| A. Yes |
| --- |
| B. No |

How many years have you suffered from diabetes?( )

Dependent on the first option of question 7.

8. Do you suffer from hyperlipidemia? ( )

| A. Yes |
| --- |
| B. No |

How many years have you suffered from hyperlipidemia? ( )

Dependent on the first option of question 8.

9. Do you smoke?

| A. I've never smoked. |
| --- |
| B. Used to smoke, now quit |
| C. Current smoking |

From the past to the present, smoking for ( ) years?

Depends on the 2nd;3rd option of question 9.

How many cigarettes do you smoke every day? ( ) (If you used to smoke and have quit, enter the number of cigarettes you used to smoke per day.)

Depends on the 2nd;3rd option of question 9.

10. Do you drink alcohol regularly? ( )

| A. never drank. |
| --- |
| B. Seldom, less than twice a week |
| C. Frequently, more than 2 times a week |

**Part II.** **Shift Work and Sleep Questionnaire**

Please read each question carefully before answering, please answer all questions to the best of your ability, and when you come across a question you don't want to answer, you can also just skip it and go to the next question.

1. In the past three months, you slept an average of ( ) hours per day? And you usually worked ( ) hours per day?

2. During the past three months, on a working day, during the day shift, did you usually take a middle nap? ( )

A. I usually take a nap. B. Generally do not nap/rarely nap C. I've been working night shifts, no naps.

3. In the past month, did you usually work during non-standard working hours (i.e., started before 7 a.m. or after 2 p.m., worked shifts, or often had to work overtime from before 7 a.m. to after 6 p.m.)?

Yes ( ) No ( )

**If yes, please start answering from question 4. If no, there is no need to answer the following questions:**

4. How many non-standard shifts do you work on average per week? ( )

5. When did you start taking non-standard classes? ( )

A. 1-12 months ago B. 1-3 years ago C. started 3 years ago

6. a. For non-rotating shifts, what time do you usually start your shift?

( )A.M. or ( )P.M.

b. For non-rotating shifts, what time do you usually leave work?

( ) A.M. or ( )P.M.

7. In the past month, while working a non-standard shift, your overall sleep time was ( )

A. adequate B. slightly inadequate C. somewhat inadequate D. very inadequate

8. In the past month, have you felt sleepy during a non-standard work shift? ( )

A. no B. slight C. rather sleepy D. very sleepy

9. In the past month, during the time you were working non-standard shifts, did you have difficulty falling asleep at bedtime? ( )

A. No B. Mildly difficult C. Quite difficult D. Very difficult

10. In the past month, have you had problems maintaining sleep while working non-standard shifts ( )

A. no problem B. minor problem C. considerable problem D. serious problem

11. In the past month, while working non-standard shifts. Have you experienced problems waking up too early to go back to sleep? ( )

A. no B. occasionally C. often D. very seriously

12. In the past month, while working non-standard shifts, what was your overall quality of sleep (regardless of how long you slept)? ( )

A. satisfied B. mildly dissatisfied C. rather dissatisfied D. very dissatisfied

13. In the past month, while working non-standard shifts, how happy were you in your waking state? ( )

A. Normal B. Mildly decreased C. Significantly decreased D. Very bad

14. How did you feel physically and mentally while awake while working non-standard shifts in the past month? ( )

A. Normal B. Mildly decreased C. Significantly decreased D. Very bad

15. In the past month, how likely are you to have dozed off during non-standard working hours? ( )

A. hardly ever B. occasionally C. moderately likely D. very likely

16.In the past month, how likely were you to doze off while driving after working a non-standard shift? ( )

A. Hardly ever B. Occasionally C. Moderately likely D. Very likely E. I don't drive

17. In the past month, how likely are you to have dozed off (not driving yourself) on the car ride to and from work after working a non-standard shift? ( )

A. hardly ever B. occasionally C. moderately likely D. very likely E. I don't need to take a car to and from work

18. During the past year, have you had at least one week when you did not work a non-standard shift (e.g., a week of vacation, or a week of standard daytime shifts)? ( )

A. Yes B. No

**If yes, please answer question 19; if no, there is no need to answer the following questions.**

19. How is your total sleep time during breaks in non-standard shifts? ( )

A. adequate B. slightly inadequate C. somewhat inadequate D. very inadequate

20. During breaks in non-standard shifts, do you feel sleepy when you are awake? ( )

A. no B. slight C. rather sleepy D. very sleepy

21. During your break from non-standard shifts, did you have difficulty falling asleep at bedtime? ( )

A. No B. Mildly difficult C. Quite difficult D. Very difficult

22. Do you have problems staying asleep during breaks in non-standard shifts? ( )

A. no problem B. minor problem C. considerable problem D. serious problem

23. When you took a break from your non-standard shift, what was your overall sleep quality (regardless of how long you slept)? ( )

A. satisfied B. mildly dissatisfied C. rather dissatisfied D. very dissatisfied

24. During your breaks from non-standard shifts when you are awake, what is your physical and mental state? ( )

A. Normal B. Mildly decreased C. Significantly decreased D. Very bad

25.On your break from a non-standard shift, how long of a delay did you experience in going to bed? ( )

A. no delay B. mild delay C. significant decrease D. very delayed

26. How likely are you to doze off or fall asleep while driving after at least two days' rest from work? ( )

A. almost impossible B. very unlikely C. moderately likely D. very likely E. I do not drive

**Part III Sexual Function**

**For the following questions, please answer based on your overall condition in the last six months**

Did you have a regular sex life? ( )

| 1. Regularly, every month or every week |
| --- |
| 1. Irregular, My wife/partner and I don't live together for a long time |
| 1. No sex |

**If you answered A, start answering the questions below, if you answered B or C, you do not need to answer questions 1-10 below.**

**Premature ejaculation diagnostic tool (PEDT)**

1. How difficult is it to delay ejaculation during sexual intercourse? ( )

A. No difficulties

B. Somewhat difficult

C. Medium Difficulty

D. Very difficult

E. Completely undelayable

2. odds of ejaculation occurring before trying to ejaculate? ( )

A. (almost) none

B. Infrequent

C. About 50%

D. Most of the time

E. Almost/always

3. Do you ejaculate when you receive very little sexual stimulation? ( )

A. (almost) none

B. Infrequent

C. About 50%

D. Most of the time

E. Almost/always

4. Is there any frustration with premature ejaculation? ( )

A. Not at all

B. Somewhat

C. General

D. very

E. Very

5. Are you concerned that the timing of ejaculation is causing dissatisfaction in your partner? ( )

A. Not at all

B. Somewhat

C. General

D. very

E. Very

**International Inventory of Erectile Dysfunction (IIEF-5)**

6. How confident are you in obtaining and maintaining an erection? ( )

A. Very low

B. Low

C. Medium

D. High

E. Very high

7. How many times have you been able to penetrate your penis when you were sexually stimulated and had an erection? ( )

A. Sexual inactivity

B. Little or nothing

C. A few times (much less than half the time)

D. Sometimes (about half the time)

E. Most of the time (much more than half the time)

F. Almost always or always

8. How many times were you able to maintain an erection after penile penetration during sexual intercourse? ( )

A. Sexual inactivity

B. Little or nothing

C. A few times (much less than half the time)

D. Sometimes (about half the time)

E. Most of the time (much more than half the time)

F. Almost always or always

9. How difficult is it for you to maintain an erection during sexual intercourse until it is completed? ( )

A. Sexual inactivity

B. Extremely difficult

C. Significant difficulties

D. Difficulties

E. Somewhat difficult

F. No difficulties

10. How often do you feel satisfied during sexual intercourse? ( )

A. Sexual inactivity

B. Little or nothing

C. A few times (much less than half the time)

D. Sometimes (about half the time)

E. Most of the time (much more than half the time)

F. Almost always or always

11. Has there been any change in the hardness of your penile erection after you have been working night/shift work for some time (months or years)? ( )

| A. No change |
| --- |
| B. Slight deterioration in hardness |
| C. Significant deterioration in hardness |
| 1. Slightly better hardness |
| 1. The hardness has improved significantly |
| 1. Always worked day shifts, not night/shift work |

12. After you have been working night/shift work for some time (months or years), does the time from penetration to ejaculation of your penis change during sex? ( )

| A. No change |
| --- |
| B. Slight reduction |
| C. Significant reduction |
| D. Slight increase |
| E. Significantly prolonged |
| 1. Always worked day shifts, not night/shift work |

**Part IV Anxiety and Depression Conditions**

**Note:** In the past 3 months, how often have the following symptoms occurred in your life? Please select the description that best matches you at the moment and fill in your score after the corresponding question.

**The Patient Health Questionnaire-9**

1. Little interest or pleasure in doing things ( )?
2. Not at all. B. Several days. C. More than half the days D. Nearly every day
3. Feeling down, depressed or hopeless ( )?
4. Not at all. B. Several days. C. More than half the days D. Nearly every day
5. Trouble falling asleep, staying asleep, or sleeping too much ( )?
6. Not at all. B. Several days. C. More than half the days D. Nearly every day
7. Feeling tired or have little energy ( )?
8. Not at all. B. Several days. C. More than half the days D. Nearly every day
9. Poor appetite or overeating ( )?

A. Not at all. B. Several days. C. More than half the days D. Nearly every day

1. Feeling bad about yourself or that you are a failure or have let yourself or your family down ( )?

A. Not at all. B. Several days. C. More than half the days D. Nearly every day

7. Trouble concentrating on things, such as reading the newspaper or watching TV ( )?

1. Not at all. B. Several days. C. More than half the days D. Nearly every day
2. Moving or speaking so slowly that others have noticed, or the opposite being so fidgety or restless that you have been moving around a lot more than usual ( )?
3. Not at all. B. Several days. C. More than half the days D. Nearly every day
4. Thoughts that you would be better off dead or of hurting yourself in some way ( )?
5. Not at all. B. Several days. C. More than half the days D. Nearly every day

**Generalized Anxiety Disorder-7**

1. Feeling nervous, anxious, or on edge ( )

A. Not at all B. Several days C. More than half of days D. Nearly every day

2. Not being able to stop or control worrying ( )

A. Not at all B. Several days C. More than half of days D. Nearly every day

3. Worrying too much about different things ( )

A. Not at all B. Several days C. More than half of days D. Nearly every day

4. Trouble relaxing ( )

A. Not at all B. Several days C. More than half of days D. Nearly every day

5. Being so restless that it’s hard to sit still ( )

A. Not at all B. Several days C. More than half of days D. Nearly every day

6. Becoming easily annoyed or irritable ( )

A. Not at all B. Several days C. More than half of days D. Nearly every day

7. Feeling afraid as if something awful might happen ( )

A. Not at all B. Several days C. More than half of days D. Nearly every day
